# Thyroid Fine-needle aspiration biopsy: an evaluation of its utility in a community setting

**DOI:** 10.1186/s40463-015-0063-9

**Published:** 2015-03-12

**Authors:** Andre R Le, Gregory W Thompson, Benjamin John A Hoyt

**Affiliations:** Memorial University Faculty of Medicine, S-1758B 300 Prince Phillip Drive, St John’s, NL A1B 3 V6 Canada; Department of Otolaryngology – Head & Neck Surgery, Zone 3, Horizon Health Network, 700 Priestman St, Fredericton, NB E3B 5 N5 Canada

**Keywords:** Fine-needle aspiration, FNA, Thyroid, Nodule, Cytopathology, Thyroid cancer, Community

## Abstract

**Background:**

Thyroid cancer rates are on the rise worldwide with over 5000 new cases estimated in Canada in 2012. The American Thyroid Association recommends the use of fine-needle aspiration biopsy (FNA) in the workup of thyroid nodules. Studies show that thyroid FNA accuracy may vary based on interpretation by cytopathologists in academic versus community centres. To date, there has been no literature published addressing the accuracy or utility of preoperative FNA in a Canadian community center. Our goals were to demonstrate the accuracy of thyroid FNA at our centre, and to compare our results to those published in the literature.

**Methods:**

Medical records for patients who underwent thyroidectomy performed by two otolaryngologists in Fredericton, NB, between September 2008 and February 2013 were reviewed. 125 patients with 197 FNAs were analyzed. Fisher’s Exact test was used to compare the malignancy rates in each FNA category, and Chi-Square test was used for FNA distribution comparison.

**Results:**

The distribution of all FNA diagnoses at our centre was as follows: 38 (19%) benign, 100 (51%) inconclusive, 8 (4%) suspicious for malignancy, 2 (1%) malignant, and 49 (25%) unsatisfactory. FNA distribution was significantly different between our centre and comparison centres (Chi-Square p < 0.05). Our malignancy rates within each category using each FNA sample as a data point were 26.3%, 29.0%, 75%, 100% and 12.2% respectively. Comparison to other community studies revealed that we have significantly higher malignancy rates with benign FNAs (Fisher’s exact p = <0.05). Analysis using our most malignant FNA data yielded similar results.

**Conclusion:**

Thyroid FNA accuracy varies between institutions, and this may affect its utility in the workup of a thyroid nodule at some centres. Expert cytopathology opinions may be an asset in interpreting FNA samples in small community centres where volumes are relatively low, however our data do not support this assertion. It is essential that physicians continue to use clinical judgment first and foremost when evaluating thyroid nodules.

## Background

It is estimated that the incidence of thyroid cancer has more than doubled in much of the developed world over the past few decades. In Canada, thyroid cancer annual incidence rates increased an average of 7% from 1998–2007. Over 5000 new cases were diagnosed in 2012 [1]. Theories have been postulated to explain this trend. It may be due to more frequent diagnostic imaging with an associated increase in radiation exposure. These tests may also be leading to the incidental discovery of earlier stage, asymptomatic thyroid cancers [1].

In the workup of thyroid nodules, one of the greatest challenges for physicians is to accurately identify which nodules have a high likelihood of harbouring malignant disease; thereby minimizing unnecessary surgical procedures and their associated risks in those with benign disease. The American Thyroid Association recommends fine needle aspiration biopsy (FNA) as a key step in the evaluation of thyroid nodules [2].

Thyroid FNA is an inexpensive, relatively safe test that may be performed in an outpatient setting. It is used to characterize thyroid nodules and to triage patients based on cytopathological results. It has been shown to demonstrate good specificity and sensitivity with respect to thyroid malignancy [3-5]. Before routinely using FNA in the workup of thyroid nodules, the malignancy rates of resected nodules were approximately fifteen percent; however, with current FNA practice these rates have reportedly increased to over fifty percent in some centres [6,7].

That said, there remain numerous limitations to FNA in the workup of thyroid nodules. For example, the skill of the aspirator and the expertise of the interpreting cytologist, both of which can vary from centre to centre, can dramatically affect accuracy of the test. Many papers have been published addressing this variability; however, the vast majority of them have been done in academic or tertiary care centres [8]. The number of studies done in a community setting is limited [8-10], and no data have been published from community centres in Canada. Given that most community centres have lower volumes than academic centres, and given that many community specialists have little sub-specialty or fellowship training, it is possible that the published accuracy of thyroid FNA may not truly reflect results seen in the community, thereby calling into question the utility of this test outside of tertiary centres.

As such, we decided to retrospectively review our experience at our Canadian community-based secondary hospital. We analyzed our thyroid FNA distribution and accuracy in the workup of thyroid nodules, and we compare these results to other international community centres [8-10] and to academic practices, including our closest major tertiary care referral centre [11].

## Methods

The medical records for all patients who underwent thyroidectomy, performed by two otolaryngologists at the Dr. Everett Chalmers Hospital in Fredericton, NB, between September 2008 and February 2013 were retrospectively reviewed. The only patients excluded from our study were those who did not have a preoperative FNA. Examples of exclusions include cases of refractory hyperthyroidism and diffuse goiter with compressive symptoms in the absence of a dominant or suspicious nodule. A statistician was consulted throughout the study design and data review.

A total of 125 patients with 197 FNAs were included for analysis. The age and gender of each patient was recorded as well as the surgical procedure performed. Initially, each FNA was treated as a separate data point and our statistical analysis was carried out. Subsequently, the data were reorganized using only the most malignant FNA for those with more than one preoperative FNA, and the statistics were repeated. There lacked uniformity in reporting from one cytopathologist to the next, and the current Bethesda criteria were not always used. As such, the FNA results were classified into one of the following categories, in increasing order of suspicion: unsatisfactory; benign; inconclusive; suspicious for malignancy and malignant.

The distribution of the FNA results across the five categories was calculated. Using Chi-square test, the distribution at our centre was compared to data published from four other centres. Each preoperative FNA result was paired with the corresponding final pathological diagnosis from the surgical specimen. Fisher’s exact test was used for comparison of malignancy rates per FNA category between centers. A p value of less than 0.05 was considered a statistically significant difference.

Ethics approval was provided by the Research Ethics Board for the Horizon Health Network.

## Results

In total, 197 thyroid FNA samples from 125 patients, 102 females and 23 males, were reviewed. Their ages ranged from 15 to 78 years with a mean age of 50.10 +/− SD 13.25 years. They went on to have a diagnostic hemi-thyroidectomy (96), a total thyroidectomy (29).

The distribution of FNA diagnoses at our centre is demonstrated in Table 1. It shows the distribution when each FNA was considered a separate data point, as well as the distribution using only the most malignant FNA in those patients with more than one preoperative FNA.

**Table 1:** **Distribution of preoperative thyroid FNA results as per diagnostic category**

**FNA Category**	**Percent of distribution**	
Unsatisfactory	49/197: 24.9%	17/125: 13.6%*
Benign	38/197: 19.2%	22/125: 17.6%*
Inconclusive	100/197: 50.8%	76/125: 60.8%*
Suspicious for malignancy	8/197: 4.1%	8/125: 6.4%*
Malignant	2/197: 1.0%	2/125: 1.6%*

*using most malignant FNA for those patients with more than one preoperative FNA.

FNA: fine-needle aspiration biopsy.

The overall rate of thyroid cancer in our study was 28.8%. Table 2 demonstrates the rate of malignancy broken down by FNA category, once again including rates considering all FNA samples as distinct data points, as well as rates using only the most malignant FNA per patient.

**Table 2:** **Malignancy rates per preoperative thyroid FNA diagnostic category**

**FNA Result**	**Malignancy rates**	
Unsatisfactory	6/49: 12.2%	2/17: 11.8%*
Benign	10/38: 26.3%	4/22: 18.0%*
Inconclusive	29/100: 29%	22/76: 28.9%*
Suspicious for malignancy	6/8: 75%	6/8: 75%*
Malignant	2/2: 100%	2/2: 100%*
Overall	26.9%	28.8%*

*using most malignant FNA for those patients with more than one preoperative FNA.

FNA: fine-needle aspiration biopsy.

Our results were then compared to three community-based centres [8-10] and one geographically close academic centre [11]. When using each FNA as a separate data point, our overall distribution across the categories was significantly different than all comparison centres (Table 3). When using only the most malignant FNA sample, the distribution at our centre remained significantly different than all but the geographically close academic centre (Table 4).

**Table 3:** **Comparison of preoperative FNA distributions between our center and other published centers shown as a percentage of all preoperative FNAs**

**FNA Result**	Blansfield et al.*	Postma et al.*	Wu et al.*	Williams et al.*	**Our center**
Unsatisfactory	6.0%	0%	9.5%	14.2%	24.9%
Benign	24.0%	28.6%	28.5%	24.2%	19.2%
Inconclusive	46.4%	48.0%	52.0%	53.6%	50.8%
Suspicious for malignancy	7.7%	6.1%	8.1%	4.4%	4.1%
Malignant	15.8%	2.0%	8.6%	3.6%	1.0%
Total	34.4%	12.0%	21.7%	28.4%	26.9%

*Chi-square p < 0.05.

FNA: fine-needle aspiration.

**Table 4:** **Comparison of preoperative FNA distributions between our center and other published studies shown as a percentage of all preoperative FNAs using the most malignant FNA per patient at our center**

**FNA Result**	Blansfield et al.*	Postma et al.*	Wu et al.*	**Williams et al.**	**Our center**
Unsatisfactory	6.0%	0%	9.5%	14.2%	13.6%
Benign	24.0%	28.6%	28.5%	24.2%	17.6%
Inconclusive	46.4%	48.0%	52.0%	53.6%	60.8%
Suspicious for malignancy	7.7%	6.1%	8.1%	4.4%	6.4%
Malignant	15.8%	2.0%	8.6%	3.6%	1.6%
Total	34.4%	12.0%	21.7%	28.4%	28.8%

*Chi-square p < 0.05.

FNA: fine-needle aspiration.

Table 5 shows the comparison of our malignancy rates to those of the four comparison centres using all FNA samples as distinct data points. Table 6 shows the same comparison using only our most malignant samples per thyroidectomy. Malignancies per FNA category yielded some interesting results. Using Fisher’s Exact test, our malignancy rates in the setting of a benign FNA are significantly higher than those at the comparison community centres.

**Table 5:** **Comparison of malignancy rates per preoperative thyroid FNA diagnostic category between our center and other published studies**

**FNA Result**	**Blansfield et al.**	**Postma et al.**	**Wu et al.**	**Williams et al.**	**Our study**
Unsatisfactory	0/11 = 0%	0/0 = 0%	3/21 = 14.2%	10/55 = 18.2%	6/49: 12.2%
Benign	3/44 = 6.8%*	0/28 = 0%*	2/63 = 3.1%*	15/94 = 16.0%	10/38: 26.3%
Inconclusive	25/85 = 29.4%	3/47 = 6%*	14/100 = 14.0%	55/208 = 26.4%	29/100: 29%
Suspicious for malignancy	8/14 = 57.1%	5/6 = 83%	10/18: 55.6%	16/17: 94.1%	6/8: 75%
Malignant	27/29 = 93.1%	2/2 = 100%	19/19 = 100%	14/14 = 100%	2/2: 100%
Total	63/183 = 34.4%	10/83 = 12.0%	48/221 = 21.7%	110/388 = 28.4%	53/197 = 26.9%

*Fisher’s exact p < 0.05.

FNA: fine-needle aspiration.

**Table 6:** **Comparison of malignancy rates per preoperative thyroid FNA diagnostic category between our center and other published studies using the most malignant FNA per patient at our center**

**FNA Result**	**Blansfield et al.**	**Postma et al.**	**Wu et al.**	**Williams et al.**	**Our study**
Unsatisfactory	0/11 = 0%	0/0 = 0%	3/21 = 14.2%	10/55 = 18.2%	2/17 = 11.8%
Benign	3/44 = 6.8%	0/28 = 0%*	2/63 = 3.1%*	15/94 = 16.0%	4/22 = 18.0%
Inconclusive	25/85 = 29.4%	3/47 = 6%*	14/100 = 14.0%	55/208 = 26.4%	22/76 = 28.9%
Suspicious for malignancy	8/14 = 57.1%	5/6 = 83%	10/18: 55.6%	16/17: 94.1%	6/8: 75.0%
Malignant	27/29 = 93.1%	2/2 = 100%	19/19 = 100%	14/14 = 100%	2/2: 100%
Total	63/183 = 34.4%	10/83 = 12.0%	48/221 = 21.7%	110/388 = 28.4%	36/125 = 28.8%

*Fisher’s exact p < 0.05.

FNA: fine-needle aspiration.

## Discussion

Thyroid cancer rates continue to rise. Furthermore, the incidence of thyroid cancer in the setting of a thyroid nodule ranges from 20% to as low as 5%. FNA has been established as the gold-standard procedure in the workup of thyroid nodules to help clinicians determine whether or not a given nodule represents malignancy. Despite being ubiquitous in the workup of thyroid nodules in North America, the accuracy and utility of this apparently simple test can vary greatly from one centre to the next. In an effort to curb some of this variability, the American Thyroid Association (ATA) has created extensive guidelines addressing the indications for FNA, and the Bethesda system has been developed and widely accepted as the manner in which FNA samples should be cytopathologically interpreted and classified.

Cibas and Ali [12] claim that the routine use of FNA in the workup of a thyroid nodule has increased the malignancy rates in resected nodules from 14% to 50%. At our centre, we anecdotally observed a very high rate of benign nodules being resected despite the use of FNA in the preoperative workup. We also felt that we were seeing an unusually high number of inconclusive FNAs, and we began to question the validity of FNA results at our institution. We hypothesized that the experience of the interpreting cytopathologists may be a contributing factor. Being a smaller secondary hospital, our volumes are relatively low compared to larger academic centres, and we do not have a dedicated head & neck pathologist reviewing all of our FNA specimens. We felt that it was possible that the relative inexperience of our pathologists when compared to the larger academic centres was leading to less accurate results from thyroid FNAs. If academic high-volume cytopathologists can produce more accurate and reliable results, then it may make sense for smaller volume centres to outsource their FNAs for interpretation by experts in centres of excellence. Our study was not powered to evaluate differences from one cytopathologist to the next.

In an effort to objectify our suspicions, we reviewed the literature and studied our data over a five-year period, comparing our results to those published. We felt it important to compare our results to those published from similar centres; however we were surprised to find no published studies addressing the accuracy and utility of FNA in a Canadian community centre. All Canadian data has been published at tertiary care hospitals. We chose our geographically closest academic centre as one of our comparison studies. We found three American studies done in community centres and we included all three in our analysis.

Our data show that only 28.8% of our resected nodules were malignant. The malignancy rates reported in the literature also seem to vary greatly, from as low as 12.0% to as high as 34.4% in the papers we chose as comparison studies. To our surprise, our overall malignancy rates were nearly identical to those published by our closest academic centre, and they were not significantly different than those published in the studies to which we compared. While it is reassuring that we aren’t resecting more benign disease than others, it is very difficult to attribute these results to FNA accuracy as the FNA is but one of many tools used in the decision to proceed with surgery. Furthermore, we are still subjecting more than 70% of our thyroidectomy patients to surgery for benign disease.

We also found that our FNA results were inconsistent; we felt that an unusually high percentage of our samples were being reported as either unsatisfactory or inconclusive. Our data clearly support this suspicion, as our distribution was significantly different than that of the comparison centres. While there are many possible explanations for this discrepancy, including the skill of both the aspirator and the interpreter, it’s difficult to clearly prove what factors are causative. But interestingly, when using only the most malignant FNA specimen in the analysis, our distribution was not significantly different than that of our closest teaching centre. This calls into question our assertion that the experience of our interpreting clinicians could be problematic.

Our most concerning finding is that 26.3% of our patients with FNAs that were reported as benign ultimately had well differentiated thyroid carcinoma. By limiting our analysis to only the most malignant specimens, that number decreases to 18.0%, still a very high false-negative rate. These results were significantly higher that those published at the other community centres, but interestingly not significantly higher than those of our closest academic centre. Wang et al., showed a statistically significant difference in false negative rates (10% vs 2%) between community and academic centers [13]. Yeh et al., demonstrated a single false negative FNA delayed treatment by an average of more than two years resulting in patients experiencing higher rates of vascular and capsular invasion [14]. Subsequently, such patients were more likely to experience persistent disease at follow-up. The discrepancy between published results is difficult to conclusively explain, but selection bias may be playing a role. In our study and in Williams et al., only patients who went on to have thyroid surgery were included. There would have been many FNAs reported as benign in patients that did not go on to surgery during the study period, and thus not captured in our data set. Those that were included in our review likely had other concerning clinical features that resulted in them having surgery despite the results. A review of all FNAs performed would be more useful in comparing malignancy rates per FNA category to published norms. That said, our data once again refute our initial assertion that academic centre cytopathologists will yield more accurate data than those at our community centre.

A big limitation of our study is that our pathology department has not yet adopted the current Bethesda classification system, and the classification can even vary slightly from one interpreter to the next. This is not unique to our institution [9,10]. To compare our results to those published, we had to re-classify some FNAs to fit into one of the chosen categories. We also had to group two categories in at least one of the comparison studies. Until all published data use uniform and current classification, comparison will remain a challenge.

This study is retrospective and is therefore potentially subject to confirmation bias. However, we did not have the authority or the resources to randomize our FNAs into two groups and outsource the “academic centre” cohort to another centre. Ideally these retrospective data support our concerns and open the door to resources for future prospective randomized research.

Our FNAs are primarily done by our radiology department under ultrasound-guidance. However, there is not a department-wide standard for technique used and not all FNAs were done with guidance. This could also be seen as a limitation. A less skilled aspirator may see a higher percentage of unsatisfactory biopsies. Biopsies taken without ultrasound guidance may not even sample the target nodule and may only get surrounding thyroid tissue. Standardized guided aspiration should be the standard at all centres, particularly when publishing data and comparing to published norms.

Lastly, volume is obviously a limitation. Our study is not adequately powered to compare to larger academic studies. However, if assessing the accuracy of FNA done in a low-volume community centre, a well-powered study is virtually impossible if done at a single centre. A multi-centre randomized trial is needed to truly assess whether low-volume centres produce less accurate FNA data in the workup of thyroid nodules.

## Conclusions

FNA remains a key tool in the investigation of thyroid nodules. Despite its use, surgeons continue to resect a very high percentage of benign disease because of uncertainty with respect to its malignant potential. Until a more accurate non-invasive diagnostic test is developed, it is important that we continue to refine our FNA technique to improve its accuracy. Regardless, clinical judgment remains of paramount importance in interpreting FNA results in the context of a given patient. Our review is the first Canadian community-based study to analyze the utility of preoperative FNA in terms of final thyroidectomy pathology. Despite our suspicion, our data do not support our hypothesis that low-volume community centre FNAs will be less accurate than those done in academic centres. That being said, it is possible that our study’s limitations prevented us from finding the real answer. Our study does show statistically significant variability in FNA distribution between our study and all three of the comparison community studies. It also shows significantly higher malignancy rates in benign FNA specimens between our centre and the three community centres. This variability may be the result of study design; of discrepancies, inconsistencies in technique or in interpretation of the FNA itself; or of difference in regional practice patterns as all three were international studies. Regardless, consistency is needed in both the aspiration and interpretation of FNAs in all centres. With consistent methodology, further prospective studies will be better able to address whether or not high-volume academic centres can produce more accurate and reliable FNA results in the workup of thyroid nodules.

## Abbreviations

FNA: Fine-needle aspiration biopsy
